# To Dr. Adams

**Published:** 1809-09

**Authors:** W. Royston

**Affiliations:** Princes Street, Cavendish Square


### To Dr. ADAMS.

Sir,

In my Report of the Progress of Medicine, for the year 1808, it was stated that the occurrence of *phthisis pulmonalis* was more frequent in the South-west of Devonshire, than in London.

I have reason to believe that this is not a correct statement; and as the ascertainment of unequivocal facts, and the communication of them to the medical world, is one of the principal objects of your Journal, I cannot doubt but you will readily admit the following explanation.

Among Dr. Woolcombe's tables there is one, the intent of which is to determine the proportional mortality from consumption. In this table is contained the deaths from consumption at Bristol, London, and Plymouth, in nearly correponding periods. And it appears that the proportional mortality from this disease at Bristol, was 1—to 2.2; at London 1—to 3.2; at Plymouth 1—to 4.28.

Admitting this calculation, "it will be found that the phthisical mortality of Plymouth has of late years been nearly one fourth less than in London, and less by nearly one half than in Bristol."

W. ROYSTON.

*Princes Street, Cavendish Square,*

*August* 15, 1809.

CRITICAL

